# Standing in Your Peer’s Shoes Hurts Your Feats: The Self-Others Discrepancy in Risk Attitude and Impulsivity

**DOI:** 10.3389/fpsyg.2016.00197

**Published:** 2016-02-19

**Authors:** Wojciech Białaszek, Piotr Bakun, Elton McGoun, Piotr Zielonka

**Affiliations:** ^1^Department of Behavior Analysis, SWPS University of Social Sciences and HumanitiesWarsaw, Poland; ^2^Department of Economic Psychology, Centre for Economic Psychology and Decision Sciences, Kozminski UniversityWarsaw, Poland; ^3^School of Management, Bucknell University, LewisburgPA, USA; ^4^Department of Econophysics, Warsaw University of Life SciencesWarsaw, Poland

**Keywords:** perspective taking, risk attitude, intertemporal choice, discounting, negotiations

## Abstract

It is often a good strategy to “stand in the other person’s shoes” to see a situation from a different perspective. People frequently attempt to infer what someone else would recommend when no advisor is available to help with a decision. Such situations commonly concern intertemporal or risky choices, and the usual assumption is that lay people make such decisions differently than experts do. The aim of our study was to determine what intertemporal and risky decisions people make when they take their own perspective, the perspective of a peer, and the perspectives of an expert or an entrepreneur. In a series of three experiments using a between-subject design, we found that taking the peer’s perspective made participants behave more impulsively and more risk aversely in relation to the participants’ own perspectives and in relation to their perceptions of experts and entrepreneurs perspectives. Taking an expert’s or an entrepreneur’s perspective did not change participants’ own intertemporal and risky decisions. We explain the findings using the risk as value and the lesser mind theories. Imagining the opponent’s perspective in a negotiation as one is advised to do might inadvertently lead to problems because we always see her as more impulsive and more risk averse than she really is. This means that taking a perspective of an expert – not a peer – would be a good way to predict what decisions our opponents make.

## Introduction

People often seek advice when making decisions. Consumers eagerly listen to the recommendations of experts before making purchases, and managers employ specialized staffers to assist them with their decision making. Unfortunately, knowledgeable advisors are not always available when they’re needed, so people instead ask themselves “What would the advisor do if the advisor were me?” In financial practice, many—if not most—decisions concern intertemporal and risky choices, and extensive research has shown that individuals tend to be impulsive and risk averse, that is, they generally prefer smaller gains now to larger ones in the future and smaller certain gains to larger riskier ones. This research aims to determine whether and how intertemporal and risky choices change when people take the perspective of others.

The problem whether risk aversion and impulsivity vary with perspective has been studied since the late 1960s, and it has been shown that when participants assume the role of advisor or are asked to make a recommendation on someone else’s behalf, they tend to recommend greater risk aversion (Clark et al., 1971; McCauley et al., 1971; Zaleska and Kogan, 1971; Kelling et al., 1976) and greater impulsivity (O’Connell et al., 2013) than they would display themselves. Possible explanations for the greater risk aversion, while taking the perspective of others are the risk as value theory (Brown, 1965; see also: Levinger and Schneider, 1969) and the lesser minds theory (Waytz et al., 2014). According to the risk as value theory, taking risk is thought to be superior behavior to avoiding risk. Thus, an advisor seeing himself or herself as superior to the advisee will assume that that person is more risk averse. According to the lesser minds theory, others have dimmer minds. So if taking risks is thought to require a superior intellect, then an advisor seeing himself or herself as smarter than the advisee will assume that that person is more risk averse. However, research conducted more recently has yielded different results regarding the influence of perspective on risk, that is, that peers tend to consider advisees to be less risk averse (Hsee and Weber, 1997). This inconsistency between more risk averse and less risk averse decisions while taking the perspective of others led us to test this issue in two ways: using a standard adjusting procedure (Du et al., 2002) and using the Holt and Laury (2002) method. The lesser minds theory may also explain greater impulsivity when taking the perspective of others. If patience is also a value (as is risk; Ainslie, 1975, 2001; Read et al., 1999) and if waiting requires a superior intellect (as does taking risks), then taking the perspective of others will make people more impulsive. Thus, when asked what peers would recommend to them, people should say that those recommendations would be worse than their own, in this case more risk averse and more impulsive. On the other hand people have high opinions of experts (Suen et al., 2014); therefore, they might say that experts’ recommendations would be better than their own, in this case less impulsive and less risk averse. However, Białek and Sawicki (2014) showed that taking the perspective of an expert shifts participants’ choices to being less impulsive but more risk averse. People often know what decisions should be made, but when it comes to acting, they behave in different ways. Normative and descriptive approaches to decision making can give different results. On one hand taking the perspective of experts might decrease risk aversion, bringing the certainty equivalent of a lottery closer to its expected value, on the other hand Białek and Sawicki’s (2014) results reveal the opposite effect. Experts, represent a normative approach to decision making, an account of how people should act. It would be interesting to test what decisions people make when they take the perspective of someone who actually makes self-controlled and risky decisions. Entrepreneurs represent a descriptive approach to self-controlled and risk seeking decisions in that they really act that way. Indeed, Macko and Tyszka (2009) revealed that in everyday business decision making in risky situations, entrepreneurs make more risky choices than non-entrepreneurs. Carland et al. (1995) and Stewart et al. (1999) showed that entrepreneurs have less risk aversion than non-entrepreneurs. Moreover, a propensity for risk seeking is posited to be one of variables underlying entrepreneurship (Sexton and Bowman, 1986; McGrath and MacMillan, 1992). Therefore, along with testing human decisions while taking the perspective of peers, we found it important to add experts as representative of the normative approach to decision making and entrepreneurs as representative of the descriptive approach. We presume that people will express less impulsivity and less risk aversion while taking the perspective of experts, who have a knowledge of how to make decisions and while taking the perspective of entrepreneurs who make self-controlled and risky decisions in practice.

Our research concerns both intertemporal and risky decisions. We compare decisions participants would make as themselves with the decisions they believe that a peer would recommend them to make and the decisions they believe that an expert or entrepreneur would recommend them to make. We suggest the following hypotheses:

Hypothesis 1 – Taking the perspective of peers will result in increasing impulsivity and risk aversion.
Hypothesis 2 – Taking the perspective of experts will result in decreasing impulsivity and risk aversion.
Hypothesis 3 – Taking the perspective of entrepreneurs will result in decreasing impulsivity and risk aversion.

## Materials and Methods

### General Method

We conducted three experiments: Study 1 addressed delay discounting (impulsivity); Study 2a addressed probability discounting (risk attitude); Study 2b also addressed risk attitude, but using a different approach than Study 2a. In Studies 1 and 2a, there were four independent conditions: (1) the “own perspective” condition (the instructions were for the participant to “choose what you would do”); (2) the “other person’s perspective” condition (the instructions were for the participant to “imagine what an average student would advise you to choose”); (3) the “expert’s perspective” condition (the instructions were for the participant to “imagine what an expert would advise you to choose”); (4) the “entrepreneur’s perspective” condition (the instructions were for the participant to “imagine what an entrepreneur would advise you to choose”). In Study 2b, we used only instructions from conditions 1 and 2.

The present research was done with hypothetical rewards rather than real ones, it having been shown that the discounting process is comparable across real and hypothetical payments (Johnson and Bickel, 2002; Madden et al., 2003; Lawyer et al., 2011; Matusiewicz et al., 2013). At the time of the study, PLN 5,000 was approximately equal to USD 1,400.

### Participants

All participants were undergraduate students at the Warsaw University of Life Sciences and at Kozminski University. All procedures were approved by the ethics committee of Kozminski University. Informed consent was gathered before the experiment. Participants were not reimbursed for their participation in the experiment.

### Data Analysis

Studies 1 and 2a used selection criteria based upon the expectation of a monotonically decreasing discounting function similar to the algorithm used by Johnson and Bickel (2008). Discounting data was considered non-systematic and excluded from further analysis if: (1) the subjective value given by a participant in the first indifference point was lower than the last indifference point or (2) the participant’s indifference points increased across consecutive delays or probabilities by more than 20% of the larger, later or the larger, more probable reward. Excluding these non-systematic cases had no effect on the directions of the effects or on the conclusions to be drawn from the studies. The selection criteria used in Study 2b are described below.

The analyses of Studies 1 and 2a were performed on the area under the discounting curve (AUC) as described in Myerson et al. (2001), which means that the analyses were model-neutral. The AUC distributions in Studies 1 and 2a and the risk seeking index distributions in Study 2b were non-normally distributed, as shown by visual inspection of the histograms and Shapiro–Wilk significance tests. Therefore, all analyses employed non-parametric statistics and medians rather than means.

## Study 1

### Participants

A total of 227 participants (91 male and 136 female from ages 19 to 52) were randomly assigned to the four conditions. After 16 were excluded for non-systematic data, the remaining 211 were distributed as follows: 52 own perspective, 53 other person’s perspective, 53 expert’s perspective, and 53 entrepreneur’s perspective.

### Procedure

Participants were tested individually in a classroom. We used a computerized procedure (standard personal computer) in which participants were asked to choose between two panels on the screen. In the first panel, the smaller adjusting amount of money was presented and in the second panel the larger, fixed amount of money was shown together with the delay. Amounts of money and delays were presented in a text format.

Participants were asked to choose between a smaller amount now and a larger amount later, from the perspective of their assigned condition. The first choice was between PLN 2,500 now and PLN 5,000 later. If the smaller amount now was chosen, the amount now was decreased; if the larger amount later were chosen, the amount now was increased. The size of the decrease or increase was PLN 5,000/2*^k^*, where *k* was the numerical order of that choice from the second (*k* = 2) through the seventh (*k* = 7). The total number of choices was six, and the indifference point was the value of the smaller amount after the final PLN 39.0625 adjustment following the sixth choice. Each adjusting value was rounded to the nearest integer. Participants made this series of choices for four different delays (1 month, 6 months, 2 years, and 5 years) to obtain four indifference points.

### Results

**FIGURE 1:**
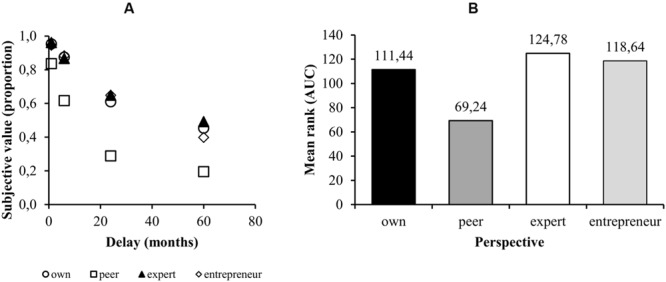
**(A)** The median indifference point (as a percentage of the delayed PLN 5,000) for each of the four lengths of delay of the larger amount for each of the conditions. **(B)** Mean ranks for the AUCs for each of the conditions.

As expected, the longer the delay of the larger amount, the smaller the amount someone prefers to take immediately (**Figure 1A**). According to a Kruskal–Wallis independent group test, there were significant differences in the AUCs for the delay discounting curves [χ^2^(3) = 26.919; *p* < 0.001]. Pairwise comparisons with a Mann–Whitney *U* test (with the Bonferroni correction) showed a significant difference between the “own perspective” and the “other person’s perspective” AUCs (*p* = 0.011, *r* = 0.343), between the “other person’s perspective” and the “expert’s perspective” (*p* < 0.001, *r* = 0.476), and between “other person’s perspective” and the “entrepreneur’s perspective” (*p* = 0.001; *r* = 0.848). There was no significant difference between the “own perspective” and the “expert’s perspective” and the “entrepreneur’s perspective” (respectively, *p* = 1 in both cases, unadjusted *p* = 0.263 and *p* = 0.546). There were also no differences between “expert’s” and “entrepreneur’s” perspectives (*p* = 1, unadjusted *p* = 0.605). These results show that discounting becomes steeper when participants take the perspective of a peer in comparison to their own, expert’s or entrepreneur’s perspectives. Mean ranks for all conditions are shown in **Figure 1B**. The descriptive statistics corresponding to raw, not ranked, data of area under the curve in different conditions are presented in **Table 1**.

**Table 1:** Median area under the curve (AUC) with interquartile range across experimental conditions during delay discounting.

	Median AUC	25th percentile	75th percentile
Own	0.660	0.300	0.814
Peer	0.347	0.176	0.521
Expert	0.646	0.458	0.884
Entrepreneur	0.634	0.425	0.810

### Discussion Study 1

Our results show that when taking the perspective of peers, one makes more present-oriented decisions than on one’s own. In other words, people perceive peer advisors as more impulsive than themselves. Our own discount functions are less steep than “taking our peer’s perspective” discount functions. These results are in line with previous research. O’Connell et al. (2013) found that people discount delayed rewards less steeply for themselves than when taking the perspective of someone they know. On the other hand, a study by Ziegler and Tunney (2012) showed that taking the perspective of another person shifts preferences toward later, larger rewards, that is, reduces delay discounting. The last seems not to be in accordance with our research, however, Ziegler and Tunney (2012) asked participants “what should the other person choose?” and not “what advice would the other person give?” A common assumption is that impulsivity is a vice whereas self-control is a virtue; therefore, when asked what one should do, people indicate less impulsive choices.

Our research shows that participants taking the perspective of an expert or entrepreneur make temporal choices very similar to the ones they would make from their own perspective. Although the mean ranks were quantitatively higher for the entrepreneur’s and expert’s perspectives than for one’s own perspective, which would indicate that taking the perspective of an expert would diminish ones impulsivity, the differences were not statistically significant. Study one has failed to confirm hypothesis 2 and hypothesis 3 regarding impulsivity.

## Study 2A

### Participants

A total of 286 participants (103 male and 183 female aged 19–48) were randomly assigned to the four conditions. After 22 were excluded for non-systematic data, the remaining 264 were distributed as follows: 64 own perspective, 67 other person’s perspective, 66 expert’s perspective, and 67 entrepreneur’s perspective.

### Procedure

The procedure was the same as in Study 1, except that all choices were between a smaller certain amount and a larger riskier amount. As in the previous study, participants were tested individually in a classroom using a computerized procedure. Participants made this series of choices for 4 different levels of risk (90, 70, 30, and 10%) to obtain four indifference points.

### Results

**FIGURE 2:**
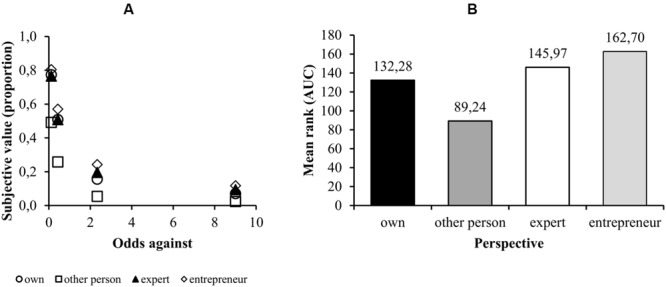
**(A)** The median indifference point (as a percentage of the uncertain PLN 5,000) for each of the four odds against the larger amount for each of the conditions (as advised by Rachlin et al., 1991) and widely used in research on discounting, the *x*-axis is in terms of the odds against the larger amount and not the probability of the larger amount). **(B)** The mean ranks for the AUCs for each of the conditions.

As expected, the smaller the probability of a larger amount of money, the smaller the amount of money a participant prefers to take with certainty (**Figure 2A**). According to a Kruskal–Wallis independent group test, there were significant differences in the AUCs for the probability discounting curves [χ^2^(3) = 21.731; *p* < 0.001]. Pairwise comparisons with a Mann–Whitney *U* test (with the Bonferroni correction) showed a significant difference between the “own perspective” and the “other person’s perspective” AUCs (*p* = 0.035, *r* = 0.277), between the “other person’s perspective” and the “expert’s perspective” (*p* = 0.001, *r* = 0.400), and between “other person’s perspective” and the “entrepreneur’s perspective” (*p* < 0.001; *r* = 0.455). These results show that when taking the other person’s perspective participants discount risky gains more steeply than when taking their own, experts or entrepreneur’s perspectives, that is, they exhibit more risk aversion.

There was no significant difference between the “own perspective” and the “expert’s perspective” or the “entrepreneur’s perspective” [respectively, *p* = 1 (unadjusted *p* = 0.263) and *p* = 0.634 (unadjusted *p* = 0.023)]. There was also no difference between “expert’s” and “entrepreneur’s” perspectives (*p* = 1, unadjusted *p* = 0.206). The mean ranks for all the conditions are presented in **Figure 2B**. The descriptive statistics corresponding to raw, not ranked, data of area under the curve in different conditions are presented in **Table 2**.

**Table 2:** Median area under the curve (AUC) with interquartile range across experimental conditions during probability discounting.

	Median AUC	25th percentile	75th percentile
Own	0.233	0.143	0.302
Peer	0.107	0.041	0.197
Expert	0.194	0.102	0.325
Entrepreneur	0.273	0.170	0.410

## Study 2B

Our results of Study 2a confirm investigations made by Brown (1965), Clark et al. (1971) and others, showing the increase of risk aversion while taking the perspective of peers. Nevertheless, our results seem inconsistent with the more recent research by Hsee and Weber (1997) indicating a decrease of risk aversion. Therefore, we decided to perform an additional experiment on risk attitude while taking a peer perspective using the Holt and Laury (2002) method.

### Participants

A total of 54 participants (23 male and 31 female aged 20 to 49 years) were randomly assigned to the two conditions as follows: 28 own perspective (the instructions were for the participant to “choose what you would do, lottery [A or B]”) and 26 other person’s perspective condition (the instructions were for the participant to “imagine what an average student would advise you to choose, lottery [A or B]”). Two participants were excluded from the analyses for irrational choices as described below.

### Procedure

Study 2b employed a traditional pen and paper questionnaire. The study was conducted individually in a classroom. Given the perspective of their randomly assigned conditions, participants were asked to choose either Lottery A or Lottery B in **Table 3** for the 10 different pairs of alternatives (participants were *not* provided with the calculations of expected values). Only very risk seeking participants would choose Lottery B in pair 1, and only very risk averse participants would choose Lottery A in pair 9. (Participants not choosing Lottery B—the only rational choice—in pair 10 were the two which were eliminated from the analysis.) The earlier in the series that participants begin to choose Lottery B, the more risk seeking they are; the later, the more risk averse.

**Table 3:** Payoff structure in Experiment 2b.

	LOTTERY A (“safe” option)				LOTTERY B (“risky” option)				Expected values (in PLN)		
	Chance	PLN	Chance	PLN	chance	PLN	chance	PLN	EV – A	EV – B	Difference (EVA – EVB)
1	10%	20	90%	16	10%	37	90%	1	16,4	4,6	11,8
2	20%	20	80%	16	20%	37	80%	1	16,8	8,2	8,6
3	30%	20	70%	16	30%	37	70%	1	17,2	11,8	5,4
4	40%	20	60%	16	40%	37	60%	1	17,6	15,4	2,2
5	50%	20	50%	16	50%	37	50%	1	18	19	–1
6	60%	20	40%	16	60%	37	40%	1	18,4	22,6	–4,2
7	70%	20	30%	16	70%	37	30%	1	18,8	26,2	–7,4
8	80%	20	20%	16	80%	37	20%	1	19,2	29,8	–10,6
9	90%	20	10%	16	90%	37	10%	1	19,6	33,4	–13,8
10	100%	20	0%	16	100%	37	0%	1	20	37	–17

*All probabilities were presented in form of chances of obtaining the reward in percentages (based on: Holt and Laury, 2002).*

### Results

The mean rank was 34.12 for the “own perspective” group and 18.88 for the “other person’s perspective” group. This was a significant difference according to a Mann–Whitney *U* test (*U* = 140,000; *p* < 0.001; *r* = 0.502). The effect size indicates the moderate impact of experimental manipulation on the dependent variable. On average, as indicated by median, participants in the “own perspective” group made 7 out of 10 choices indicating risk-taking, and participants in the “other persons” group made 5 out of 10 choices indicating risk. The results show that participants in the other person’s perspective group were more risk averse.

### Discussion Studies 2a and 2b

Using the adjusting procedure in Study 2a, we found that taking the perspective of a peer results in an increase of risk aversion in relation to taking one’s own perspective. These results contradict those of Hsee and Weber (1997), which showed a decrease of risk aversion when taking a peer’s perspective. In order to explain such a self-others discrepancy in risky choices, we conducted Study 2b using a different method – the Holt and Laury (2002) approach—which study gave the same results as Study 2a. Since the results obtained by both methods are congruent, we regarded replication of other conditions from Study 2a, using the Holt and Laury (2002) method, as redundant. Taking the perspective of a peer increases risk aversion in relation to taking one’s own perspective. Our results do not support those of Białek and Sawicki (2014), who found that when participants took the expert’s perspective, they exhibited greater risk aversion. Instead, our research shows that taking the perspectives of experts and entrepreneurs does not change the appraisal of risky payoffs in comparison to one’s choices.

## General Discussion

In a business context, negotiations demand an assessment of the opponent’s position, and in private life, it is always advisable to imagine the point of view of a partner. The main aim of our research was to test how a shift of perspective affects intertemporal and risky decisions.

People commonly regard that recommendations gave by their peers are worse than their own, in this case more risk averse and more impulsive. On the contrary, having high opinions of experts (Suen et al., 2014), people may regard experts’ advice as better than their own. Entrepreneurs especially exhibit both high self-control and low risk aversion (Carland et al., 1995; Stewart et al., 1999; Macko and Tyszka, 2009). Experts represent a normative approach to a decision making whereas entrepreneurs represent a descriptive approach to self-controlled and risk seeking decisions in that they really act that way.

Our Hypothesis 1 (Taking the perspective of peers will result in increasing impulsivity and risk aversion) has been confirmed, whereas our Hypothesis 2 (Taking the perspective of experts will result in decreasing impulsivity and risk aversion, and Hypothesis 3 (Taking the perspective of entrepreneurs will result in decreasing impulsivity and risk aversion) have not been confirmed. When taking the perspective of a peer, participants showed greater impulsivity and greater risk aversion. When taking an expert’s or entrepreneur’s perspective participants did not change intertemporal and risky choices from those they would make on they own. However, when taking the perspective of an expert or entrepreneur participants behaved less impulsively and more risk seeking than when taking the peer’s perspective.

Previous research shows that people discount delayed rewards more steeply (are more impulsive) when taking the perspective of peers (O’Connell et al., 2013), which is consistent with our results. There has been almost no research on intertemporal choices while taking the perspective of experts. Białek and Sawicki (2014) showed that taking the perspective of an expert shifts participants’ choices to being less impulsive. Our study did not show this effect, and we did not observe any difference in participants’ intertemporal choices while taking the perspective of an entrepreneur.

Risk attitudes while taking the perspective of others have generally been shown to change in the direction of greater risk aversion (Brown, 1965; Clark et al., 1971; McCauley et al., 1971; Zaleska and Kogan, 1971; Kelling et al., 1976). Some researchers (Hsee and Weber, 1997), however, obtained the opposite results. This marked inconsistency led us to conduct the research on risk attitude while taking a peer perspective in two different ways: using a standard adjusting procedure (Du et al., 2002) and using the Holt and Laury (2002) method. Regardless of the method used, it turns out that taking the perspective of peers evokes greater risk aversion.

Our research shows no significant change in risk attitude when comparing one’s own choices and those made while taking the perspective of an expert or even an entrepreneur. We did not confirm the finding of Białek and Sawicki (2014), who showed that taking the perspective of an expert evoked greater risk aversion. They raise an important question whether taking the perspective of an expert is a proper tool of debiasing people. As they show, the increase of self-control can be regarded as debiasing, but the increase of risk aversion cannot. It would be interesting to investigate other perspectives that might decrease people’s risk aversion.

Why do people imagine choices of peers being different from their own (more impulsive and more risk averse), while there is no difference in impulsivity and risk attitude when taking the perspective of an expert or entrepreneur? Risk as value theory (Brown, 1965) ascribes a positive attitude toward risky choices; taking risk deserves respect and approval (Shapira, 1995). The lesser mind theory states that people view others’ minds as lesser than their own (Waytz et al., 2014), generally regarding themselves as better than others in many areas (e.g., Svenson et al., 1985). Perceiving others as having dimmer minds, people conclude that others will not possess the virtues of risk taking and patience. People have high opinions both of experts (Suen et al., 2014) and of themselves, so we might have expected there to be no difference between what participants would do themselves and what they think experts (or entrepreneurs) would recommend that they do. Furthermore, individuals want to maintain high self-esteem (Crocker and Park, 2004) and consistent feelings of personal worth (Brown, 1986; Brown et al., 1988).

Various tasks people face in their lives can be divided into two categories: competence tasks and moral tasks (Wojciszke, 2005). Medical doctors and auto mechanics seem to deal mostly with competence tasks, whereas psychotherapists deal with moral ones. The latter do not strictly follow objective criteria in their work and seem to rely in some part on intuition. In our opinion, people perceive their own decisions in competence fields as worse than those made by experts, but they think their moral abilities are equal to those of experts. Resisting temptation and sticking to long-term goals is regarded as a virtue (Ainslie, 1975, 2001; Read et al., 1999). Similarly, risk taking is treated as a value (Brown et al., 1988). Thus intertemporal and risky decisions seem to belong to the moral category resulting in a finding that when taking the perspective of an expert or entrepreneur, there is no significant change in people’s preferences toward either delayed or risky payments.

Credible experts are perceived as holding opinions consistent with one’s own (Raviv et al., 1993; Bonaccio and Dalal, 2006) and recommending risky actions (Barnoy et al., 2010; Bar-Tal et al., 2013). This may be an explanation why people do not change their choices while taking the perspective of an expert or entrepreneur, believing that experts should behave just like themselves.

Taking the perspective of a peer does not reflect the risky and intertemporal decisions people really make for themselves. Actually being able to take someone else’s perspective in a negotiation may be an illusion. People cannot put themselves in their opponents’ shoes, seeing them as are more risk averse and impulsive (and less capable of performing the feats of making good decisions) than they really are. On the other hand, taking the perspective of an expert or entrepreneur does not change people’s own risky or intertemporal decisions. This means that taking a perspective of an expert – not a peer – would be a good way to predict what decisions our opponents make.

## Author Contributions

All authors contributed to the presented work. Authors took part in drafting or revising it critically for important intellectual content and approved the final version to be published. Also all authors ensured that questions related to the accuracy or integrity of any part of the work are appropriately investigated and resolved. Especially: WB, PZ: substantial contribution to the conception and design of the work, acquisition, analysis, interpretation of data for the work; PB: acquisition of the data; EM: interpretation of data.

## Conflict of Interest Statement

The authors declare that the research was conducted in the absence of any commercial or financial relationships that could be construed as a potential conflict of interest.
